# Estimate the prevalence of daily caffeine consumption, caffeine use disorder, caffeine withdrawal and perceived harm in Iran: a cross-sectional study

**DOI:** 10.1038/s41598-024-58496-8

**Published:** 2024-04-01

**Authors:** Fatemeh Abdoli, Mohammadreza Davoudi, Fereshte Momeni, Farhang Djafari, Behrooz Dolatshahi, Samaneh Hosseinzadeh, Hajar Aliyaki, Zahra Khalili

**Affiliations:** 1 Substance Abuse and Dependence Research Center, University of Social Welfare and Rehabilitation Sciences, Tehran, Iran; 2 Department of Clinical Psychology, School of Behavioral Sciences, University of Social Welfare and Rehabilitation Sciences, Tehran, Iran; 3 Department of Community Nutrition, School of Nutritional Sciences and Dietetics, Tehran University of Medical Sciences (TUMS), Tehran, Iran; 4 Biostatistics Department, University of Social Welfare and Rehabilitation Sciences, Tehran, Iran

**Keywords:** Addiction, Caffeine, Depression, Caffeine use disorder, Psychology, Comorbidities

## Abstract

One of the informal diagnoses in DSM-5 is Caffeine Use Disorder (CUD). CUD and high levels of caffeine consumption could impact mental health conditions. This study aimed to estimate the prevalence of CUD, caffeine consumption, caffeine-related harms, and related psychiatric symptoms in Iran. A cross-sectional survey with a convenience sample of 1228 adults were conducted in Iran. Caffeine consumption was assessed across 20 products in Iran. Caffeine Use Disorder Questionnaire (CUDQ), Caffeine Withdrawal Symptoms Questionnaire (CWSQ), 14-item Caffeine-related Harm Screening (CHS), and Symptom Checklist-25 (SCL-25) were used in the present study. We used SPSS (desktop version 26.0) to analyze the data using descriptive statistics, chi-square, and the least significant difference (LSD) post hoc test. The daily average caffeine consumption was 146.67 mg. The prevalence of CUD and caffeine withdrawal (C.W.) were estimated at 19.5% and 46.62%, respectively. Also, 12.9% of responders received CUD and C.W.s simultaneously. The prevalence of CUD was higher in men than females (25.08% vs. 13.93%). 95% of participants (n = 1166) reported using at least one caffeine product yesterday. Moreover, the most reported caffeine-related harms were the desire for sugar (42.9%), insomnia (39.3%), and caffeine dependence (38.3%). Age significantly correlates with CUD (− 0.07) and daily caffeine intake (0.08). Moreover, all SCL-90 subscales had a significant correlation with daily caffeine intake. Finally, responders at younger ages reported higher levels of CUD and caffeine consumption than older adults(*P* < 0.05). High rates of C.W. and CUD in the Iranian population suggest that it is necessary to develop evidence-based treatments.

## Introduction

Caffeine is the most commonly consumed psychoactive substance worldwide^1^. Caffeine consumption increases alertness and concentration^2^, cognitive performance^3^, and physical strength^4^.

The evidence indicates the effectiveness of this substance on Alzheimer’s, Parkinson’s, and persistent depression^5,6^. Also, some epidemiological studies showed that consuming caffeine products at normal levels can protect against dementia, non-alcoholic fatty liver disease, metabolic syndrome, and type 2 diabetes^7,8^.

However, caffeine consumption is associated with potential adverse physical and psychological effects. For example, caffeine is contraindicated for those with gastrointestinal problems, urinary incontinence, insomnia, and anxiety, and its use during pregnancy is associated with weakness^9^.

Caffeine in higher doses can cause symptoms of failure and intoxication (such as digestive discomfort, insomnia, and restlessness^10^. Additionally, excessive caffeine consumption leads to symptoms that overlap with many psychiatric disorders. Excessive caffeine consumption is associated with exacerbation of psychotic symptoms and aggression, increased incidence of stroke, and worsening of anxiety symptoms and sleep disorders^11–13^. Also, some caffeine users become dependent on this substance and show symptoms of withdrawal and deprivation^13^.

In this regard, the diagnosis of Caffeine Use Disorder (CUD) is mentioned in DSM-5, in part III (as a suggestion for further study). Recent studies have shown that in different countries, between 8 and 20 percent of people have a caffeine consumption disorder. CUD, especially in sensitive individuals, can cause symptoms of "caffeinism." Symptoms of this condition include anxiety, restlessness, nervousness, grouch, insomnia, excitement, psychomotor agitation, and inappropriate flow of thought and speech^14^.

High doses (greater than 300 mg) of caffeine regularly have been shown to delay recovery in bipolar patients and cause mood swings and anxiety^15,16^. Caffeine consumption disorder can even cause manic episodes and psychotic symptoms in those who have not previously suffered from these disorders^17,18^.

One country that exports tea (one of the most common caffeinated substances) is Iran. Due to the legal ban on the consumption of alcoholic beverages, caffeinated substances are the only available option for serving in parties and other life situations. Despite these cases, no study has investigated use patterns and the prevalence of CUD. Also, the prevalence of psychiatric disorders related to it is still unknown^18,19^.

Awareness of these cases can help diagnose, treat, and intervene to withdraw CUD and improve individual and social interventions by identifying related risk factors. Therefore, as a cross-sectional study, the current study aims to investigate the prevalence of CUD and its relationship with psychiatric symptoms and demographic features in the Iranian population.

## Method

### Study design

The study is cross-sectional and descriptive-analytical. The ethics code of this research (IR.USWR.REC.1401.037) was received from the Tehran University of Welfare and Rehabilitation Sciences in May 2022.

### Participants and process

This research data was collected between June and August 2022 through Paper-and-Pencil questionnaires in Tehran, Iran. In the first step, an announcement about the study prepared and shared on the most common social media in Iran (include Instagram, Telegram, and Tiktok). In the advertisement, researchers of the current study provided some information about the study and its process. They then asked volunteers to go to an address based on their accessibility from four places: Nezam Mafi Rehabilitation Center (located in the west of Tehran), Rofeyde Rehabilitation Hospital (located in the north), Razi Psychiatric Hospital (located in the south), and Asma Rehabilitation Center (located in the east). All of these centers are affiliated with the University of Social Welfare and Rehabilitation Sciences, Tehran, Iran, which provided ethical approval and supervision for the study. In each center, one of the authors of the current study worked as a psychologist and was ready to gather data.

In the current study, participants were asked to respond to the caffeine they consumed based on a checklist for the previous day. We chose the last day to prevent recalling bias and memory problems.

Also, they responded to a demographic checklist, the caffeine use disorder questionnaire, the caffeine withdrawal symptoms questionnaire, the 14-item caffeine-related Harm screening, and the symptom checklist-25.

Before the main study, we conducted a pilot test with 25 psychology students to evaluate the comprehensibility and clarity of the questionnaire. Their feedback allowed us to refine and improve the questionnaire, addressing any potential ambiguities that could lead to recall bias.

This research was survey research based on quota sampling. According to the following formula, the sample size estimated was 1473. However, we added 8% to that to reduce potential bias in the self-report scale, so the final sample size was 1589 participants.

*n* is the required sample size.

*Z* is the Z-score corresponding to the desired confidence level, which we consider 99% (Z = 2.576).

*p* represents the estimated prevalence of psychiatric disorders in the population. According to a study conducted in New Zealand, which showed a prevalence of 20% for caffeine use disorder, we consider 0.2 for *p*^20^.

d is the desired margin of error, which we considered 0.03.

$$n = \frac{{z_{{1 - {a \mathord{\left/ {\vphantom {a 2}} \right. \kern-0pt} 2}}}^{2} \times p\left( {1 - p} \right)}}{{d^{2} }}$$

### Translation and cross-cultural adaptation process

The current study was the first utilization of the Caffeine Use Disorder Questionnaire (CUDQ) and Caffeine Withdrawal Symptoms Questionnaire (CWSQ) scales within the Persian context. The authors used the American Psychological Association (APA) translation and cross-cultural adaptation criteria.

*Forward translation*

Both questionnaires, originally in English, were translated into Persian (Iranian language) by two psychologists proficient in English. They generated Form X for each scale. Additionally, two English teachers with over 15 years of experience (without knowledge of psychology) created Form Y for both scales.

*Translation synthesis*

After the initial translations, a meeting was convened with the all translators to consolidate their versions into a unified questionnaire labeled as Z for each scale.

*Backward translation*

Two translators from England, proficient in Persian but lacking training in behavioral sciences and access to the Caffeine Use Disorder Questionnaire (CUDQ), Caffeine Withdrawal Symptoms Questionnaire (CWSQ), or comparable measures, translated the synthesized Persian version back into English. The resulting final forms are designated as F forms.

*Expert committee*

A committee of two writers and translators with expertise in methodology, psychometrics, epidemiology, and biostatistics was formed to compare the back-translated versions with the original questionnaires. Identifying and rectifying translation errors, their goal was to enhance the comprehensibility and appropriateness of the pre-final Persian versions for a broader Persian audience.

*Pilot testing*

In a preliminary study, 60 psychology students assessed the comprehensibility and clarity of the Persian versions. They were instructed to utilize the tool and highlight any ambiguous items in their responses. Subsequently, the final versions were prepared based on their feedback.

### Measures

- **Demographic information questionnaire and caffeine consumption checklist**: This questionnaire was designed to collect individual information regularly and determine caffeine use patterns and the amount of caffeine consumption. This checklist is a researcher-made checklist under the guidance of mentors and advisors. At first, to measure the amount and type of consumption of each caffeinated product, with the help of a nutritionist, a university professor in the field of psychology, an expert in the field of addiction, and a marketer in the field of sales of caffeinated products, a checklist of caffeinated products that are available in Iran was prepared. Demographic questions included information on age, gender, socioeconomic status, employment status, and marital status using standardized questions. Also, the questioned person specified the number of times of consumption of each caffeinated product in the questionnaire based on day, week, month, or year. The frequency of caffeine consumption in this questionnaire is in the form of a spectrum that started from “I do not eat” and continued to “once a year.” The participant could indicate the amount of daily consumption (if any) in the form of an open-ended question. In this questionnaire, 20 caffeinated substances common in Iran were asked. According to to previous studies, low consumption is defined as less than 200 mg/day, moderate consumption is considered in the range of 200–400 mg/day, and high consumption is defined as exceeding 400 mg/day^20^.
- **Caffeine use disorder questionnaire (CUDQ):** This questionnaire was designed by Ágoston, Urban, Richman, and Demetrovics in 2018^21^. This questionnaire is a 10-item scale based on the nine criteria proposed for CUD in DSM-5, which is accompanied by an additional item related to the suffering caused by the symptoms and the severity of the symptoms of caffeine consumption during the last 12 months. It is measured on a 4-point Likert scale (0 = never, 3 = always). To meet the criteria for CUD, participants must endorse at least the first three items. Cronbach's alpha of this questionnaire was measured outside of Iran in research by Booth, Saxton, and Rodda, which is equal to 0.82^20^. In this research, this questionnaire was implemented as a pilot. A total of 152 community members completed the questionnaire at the beginning of the research, and its internal consistency was equal to 0.770. Also, 30 people completed the questionnaire again in two weeks, and its test–retest value was 0.94 with a 95% confidence interval.
- **Caffeine withdrawal symptoms questionnaire (CWSQ):** This questionnaire was designed by Juliano, Huntley, Harrell, and Westerman in 2012^22^. This scale measures five withdrawal symptoms within 24 h after a sudden reduction in caffeine consumption on a yes–no basis). Cronbach's alpha is reported as 0.78. In the present research, to check the repeatability of this questionnaire, 30 people answered the questions twice in two weeks. Its test–retest value was equal to 0.98 with a confidence interval of 95%.
- **14-item caffeine-related harm screening (CHS):** This questionnaire was designed by Booth, Saxton, and Rodda in 2020^20^. This tool measures the amount of damage in the last 12 months with the answer (yes/no). The items measure the following: physiological harms (e.g., headache, insomnia, fatigue, stomach discomfort), psychological factors (e.g., feeling dependent), and other harms such as high cost and tooth stains, which do not necessarily lead to clinical disorders. The internal consistency of this questionnaire was evaluated, and its Cronbach's alpha was equal to 0.86. In the present research, first, this questionnaire was translated with the help of several experts. To check the repeatability of this questionnaire, 30 people answered the questions twice in two weeks. Its test–retest value was equal to 0.90 with a confidence interval of 95%.
- **Symptom checklist-25 (SCL-25)**: This checklist was designed by Najarian and Davoudi in 2001^23^. This questionnaire is a 25-item self-report scale that is a shortened form of the SCL-90, most of which is taken directly from the Hopkins Symptom Checklist. This checklist is a unique collection for evaluating psychological symptoms and psychological distress that measures nine different psychological dimensions, including physical complaints, obsessions, and compulsions, sensitivity in mutual relationships, depression, anxiety, aggression, phobia, paranoid thoughts, and psychosis, five-point scale from zero (none) to four (severe)^24^. In Najarian’s research (2001), it was observed that this checklist has a significant correlation with its original form (SCL-90), and as a result, it is a valid tool for measuring the symptoms of mental disorders.

### Statical analysis

All analyses were conducted using the Statistical Package for the Social Sciences (SPSS) software, version 26. Tests related to continuous variables, such as independent t-groups and one-way analysis of variance (ANOVA), were employed to describe the data. For discrete variables, the chi-square test was utilized. Additionally, the least significant difference (LSD) post hoc test was applied to compare quantitative scores between different groups.

### Ethical approval

The ethics committee of the University of Social Welfare and Rehabilitation Sciences, Tehran, Iran (IR.USWR.REC.1401.037), approved this study. Moreover, written informed consent was achieved from all patients before the research procedures were operated. In addition, we informed them that they could leave the project whenever they wanted without any consequences. All procedures were carried out in compliance with the ethical rules and regulations or the Helsinki Declaration.

## Results

### Participant's characteristic

One thousand five hundred eighty-nine participants consented to take part in the survey. Given the focus of the paper, we excluded participants who did not complete the following measurement tools: caffeine consumption checklist (n = 77), CUD (n = 179), caffeine withdrawal (n = 29), and caffeine-related harm (n = 76). A total of n = 1228 respondents (77.28% of the initial sample) were included for analysis. As shown in Table 1, respondents were in middle age (median = 34, range 17–59), and nearly half of the sample were female.

**Table 1:** Caffeine consumption levels, CUD, and caffeine withdrawal, by demographic characteristics (n = 1228 unless stated ^a^).

Demographics	Total sample	Level of daily caffeine consumption			CUD (n = 1223)^b^	Caffeine withdrawal	Combined CUD and Caffeine withdrawal
		Low consumption < 200 mg/day (n = 897)	Moderate consumption 200–400 mg/day (n = 226)	High consumption > 400 mg/day (n = 105)			
Age	35.49 ± 11.70	35.77 ± 12.09	36.21 ± 9.57	31.51 ± 11.81	36.92 ± 10.56	35.77 ± 11.96	34.30 ± 10.59
Gender (1227 responders) (n%)							
Male	617 (50.2%)	368 (59.64%)	151 (24.47%)	73 (11.83%)	86 (13.93%)	322(52.18%)	68 (11.02%)
Female	610 (49.7%)	510 (83.60%)	75 (12.29%)	32 (5.24%)	153 (25.08%)	245(40.16%)	90 (14.75%)
Employment status, n (%)							
Employed	676 (55.18%)	463 (68.49%)	170 (25.14%)	43 (6.36%)	96 (14.2%)	321 (47.48%)	56 (8.28%)
Unemployed	67 (5.46%)	45 (67.16%)	3 (4.47%)	19 (28.35%)	21 (31.34%)	46 (68.65%)	18 (26.86%)
Other (e.g., student)	485 (39.59%)	389 (80.20%)	53 (10.92%)	43 (8.86%)	122 (25.15%)	220 (45.36%)	84 (17.73%)
Marital status							
Single	642 (52.22%)	473 (73.67%)	95 (14.79%)	74 (11.52%)	111 (17.26%)	310 (48.28%)	89 (13.86%)
Married or in a relationship	586 (47.78%)	424 (71.84%)	131 (22.35%)	31 (5.29%)	128 (21.84%)	257 (43.85%)	69 (11.77%)

^a^ = Due to rounding, not all within-group percentages add up to 100%. ^b^ = To meet DSM-5 criteria for CUD, respondents had to endorse CUDQ items 1–3, ^total^ number = 1227.

The results showed that the average caffeine consumption in the Iranian population was 146.67 mg. The prevalence of CUD and caffeine withdrawal was 19.5% and 46.62%, respectively. Also, 12.9% of participants showed a simultaneous diagnosis of CUD and caffeine withdrawal. Moreover, the results showed that there are significant age differences between different categories of caffeine consumption (low, medium, and high doses) (F = 6.81, *P* < 0.01). LSD results showed that the group consuming high caffeine doses was significantly younger. However, there is no significant difference in age between low-dose and medium-dose users.

### Sociodemographic differences in CUD severity and daily caffeine consumption

Table 2 shows the diversity of caffeine consumption in different age groups, genders, and education levels. Also, using the quantitative version of the CUD scale, CUD severity has been compared in different subgroups. All caffeine users responded to the CUD severity scale (based on Likert; see procedures section for more information). Table 2 shows that men's daily caffeine consumption rate is significantly higher than women. However, CUD severity was not significantly different between women and men. Also, the chi-square test results showed that the prevalence of CUD in women (25.08%) is significantly higher than in men (13.93%).

**Table 2:** Sociodemographic descriptions regarding subgroups.

Variable		Caffeine consumption		CUD severity		CUD diagnosis 5	
		Mean (S.D.)	*p*	Mean (S.D.)	*p*	N (%)	*P*
Gender	Female (n = 617)	119.53 (121.83)	< 0.01	7.32 (5.2)	0.32	153 (25.08%)	< 0.01
	Male(n = 610)	183.51 (190.22)		7.04 (5.29)		86 (13.93%)	
Age	Young adults (18–24) (n = 273)	185.5 (207.93)	< 0.01	7.64 (6.21)	< 0.01	35 (12.91%)	< 0.01
	Adulthood (25–43) (n = 623)	146.76 (147.49)		7.47 (4.9)		144 (23.18%)	
	Middle age (44–64) (n = 333)	131.48 (142.2)		6.23 (4.7)		60 (18.12%)	

We used the International Encyclopedia of the Social & Behavioral Sciences guidelines, and Geifman et al. proposed age stages to define the age in categorical data^25,26^.

The results showed a significant difference in daily caffeine consumption between different age groups. LSD results showed that young adults reported significantly higher levels of caffeine consumption than the other two groups. Also, there was no significant difference in caffeine consumption between Adulthood and Middle age. Also, there were differences between different age groups in CUD severity. There was no significant difference in CUD severity between young adults and adulthood. However, young adults showed higher levels of CUD severity than middle age. Regarding CUD diagnosis, there was a significant difference between different age groups.

### Caffeine consumption by product

**Figure 1:**
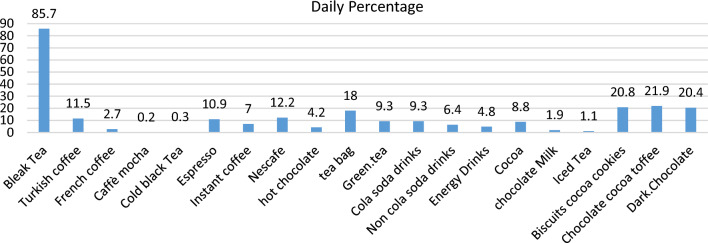
The percentage of caffeine consumption on the previous day by the participants.

Among the 1228 participants, 1166 (95%) reported consuming at least one caffeinated substance the previous day. In Fig. 1, the consumption amount of each caffeinated substance on the last day is listed separately by consumption. As shown in this figure, brewed tea is the most used caffeinated substance in Iran, with a rate of 85.7%. Caffeinated toffee, biscuits, and dark chocolate were placed in the next positions.

### Prevalence of experienced caffeine-related harms

Regarding self-reported symptoms of the harms of caffeine consumption, 1220 people responded to the caffeine harms questionnaire. Of these subjects, 1062 (87%) experienced at least one symptom related to perceived harm from caffeine. As shown in Table 3, the most common symptoms were increased desire to consume sugary substances, insomnia, and caffeine dependence, respectively.

**Table 3:** Prevalence of experienced caffeine-related harms.

Reported harm	Percent	N
Fatigue	25.4	312
Irritability	30.6	376
Reduced functioning	29.9	367
Headache	36.8	452
Insomnia	39.3	482
Feel dependent	38.3	470
Upset stomach	32.0	393
Dehydration	28.7	352
Muscle tremors	14.9	183
Increased heart rate	19.8	243
Skin problems	14.3	175
Teeth stains	28.6	351
Desire for sugar	42.9	527
Spending too much	19.7	242

### Associations between CUD and clinical variables

The results showed that the prevalence of caffeine consumption disorder and caffeine withdrawal was 19.5% and 46.62%, respectively. Table 2 examines the correlation between clinical variables and the intensity of caffeine consumption. As shown in Table 3, age significantly correlates with the daily amount of caffeine consumption (positively) and CUD (negatively). Also, with the increase in daily consumption, the severity of CUD was significantly increased in people who received a diagnosis of CUD. Among the psychiatric symptoms, depression had no significant relationship with the amount of caffeine consumption. However, a significant and linear relationship was found between the severity of CUD and depression. Regarding other variables, Table 4 provides detailed information.

**Table 4:** Pearson correlation among variables.

	Age	CUD	Somatization	Obsessive–compulsive	Interpersonal sensitivity	Depression	Anxiety	Hostility	Phobia	Paranoid ideation	Psychoticism
Daily caffeine intake	0.086**	0.246**	0.03	− 0.035	− 0.026	0.043	0.006	0.275**	0.225**	− 0.075**	− 0.096**
CUD severity	− 0.070*	1	0.353**	0.211**	0.133**	0.296**	0.343**	0.164**	0.337**	0.433**	0.363**

a = SCL-90 subscales, **correlation is significant at the 0.01 level, *correlation is significant at the 0.05 level.

## Discussion

This study aims to investigate caffeine use patterns, CUD, CWSs, caffeine-related harm, and their relationship with psychiatric symptoms, taking into account a wide range of demographic information among Adults in Iran. Among the participants, 19.5% met the criteria for CUD, 46.62% met the criteria for CWS, and 87% had reported at least one caffeine-related harm in the past year.

The highest average consumption among the daily products was related to tea, toffee, biscuit, and dark chocolate, consumed by 85.7, 21.9, 20.8, and 20.4 percent of the participants, respectively. Also, 18.7% of participants consume > 400 mg/day caffeine. In Iran, tea is cultivated in the country’s northern regions, including Lahijan, which has reduced the cost of tea production in Iran. In addition, tea consumption has long been considered the leading custom of getting together, and few people in Iran do not drink a cup of tea in the morning^27^. Although many people often mention its taste as the reason for consumption, the preference for the bitter and certainly bad taste of caffeine is probably due to the positive effects of this psychoactive substance and its association with mood changes caused by its consumption^28^.According to the results, the age of the participants who had the highest amount of caffeine consumption (> 400 mg/day) was lower than the other groups and, unlike the study of Frary, Johansson and Wang^29^, the correlation between age and caffeine consumption was negative. This issue can be because, nowadays, younger people usually consume caffeine from different sources and new products such as energy drinks, dark chocolate, and carbonated drinks. Furthermore, with age, due to some physical issues such as blood sugar, caffeine consumption will generally be limited to tea and coffee. The young generation, like students and athletes, consumes caffeine to feel alert, increase sociability, increase physical energy, improve mood, and reduce stress. Energy drinks, a new popular caffeinated beverage, are also often consumed to boost energy or combat insufficient sleep^30^. Of course, marketing some caffeinated products, such as energy drinks, soft drinks, and ready-to-drink alcoholic beverages, which often target specific populations such as young adults and teenagers, is not without influence^30,31^.

Although nutritional knowledge may influence caffeine consumption^32^, beliefs, experiences, and information from peers may change how consumers interpret their feelings^33^.

As mentioned, among participants, 19.5% met the criteria for caffeine use disorder. This finding is consistent with the results of the research conducted by Booth, Saxton, and Rodda (20%) in 2020 in New Zealand^20^, But these results are higher than the results of Sweeney et al.'s research (8%) ^13^and Ágoston’s research (13.5%) ^21^. At first, People who consume caffeine in a problematic way may be unaware of its physical and psychological consequences or cannot attribute the side effects they experience to caffeine consumption. Also, it has been shown in some research^34^ that health-related students reduce their caffeine consumption after being aware of the consequences of this substance. Other people may use caffeine as a coping mechanism and an addictive pattern to face their existing conditions, leading to excessive consumption of these products^35^. In this regard, considering the opinion of addiction experts is an important supplement to evaluate the clinical significance of CUD.

Continuous consumption of even less than 100 mg of caffeine, or 3–7 days of high doses of caffeine, can also lead to caffeine withdrawal syndrome, so that even a short abstinence, such as missing a cup of coffee or tea in the morning, can lead to significant unpleasant effects, that drinking even low doses of caffeine suppresses these symptoms^36^. Since about 86% of the participants consumed a caffeinated product daily, it seems normal that 46.62% met the criteria for CWSs. These results are similar to those of a New Zealand study^20^, in which 30 percent of participants had experienced caffeine withdrawal symptoms.

Among participants, 87 percent reported at least one caffeine-related harm in the past year. These results are similar to those of a New Zealand study^20^, in which 85 percent of participants had experienced at least one caffeine-related harm in the past year. According to the results, the participants' most experienced caffeine-related harm was increased sugar cravings (42.9%) and sleep difficulties (39.3%). Although consumption in low to moderate doses leads to pleasant sensations, higher doses taken at once or for short periods can cause or exacerbate restlessness, insomnia, nervousness, and anxiety^37^. Furthermore, most evidence shows that caffeine alone increases the adverse effects of glucose metabolism and reduces insulin sensitivity^38^. Caffeine’s reputation as a stimulant that can compensate for fatigue-related deficits means that the substance is often consumed by tired people, even though caffeine itself has been implicated in causing fatigue in the first place^39^. The “coffee cycle” phenomenon can partially explain this: feeling tired in the morning causes more caffeine consumption, which is associated with disruption of subsequent sleep patterns^40^.

The results of Bergin and Kendler’s study in 2012 showed that generalized anxiety disorder, phobia, and major depressive disorder have common genetic factors with caffeine consumption, and their genetic correlation was estimated at 0.48, 0.25, and 0.38, respectively^41^; in this research correlation between CUD severity and anxiety, phobia, and depression symptoms was 0.34, 0.33 and 0.26 respectively. Hearn and his colleagues also observed in 2020 that the daily consumption of caffeine among the general population is significantly higher than that of the patient population, but the rate of poisoning caffeine doses was higher among the clinical population^42^. Such research can lead us to conclude that significant common genetic and environmental factors between psychiatric disorders and caffeine phenotypes may help us in the etiology of the coexistence between these phenotypes.

## Limitations and future directions

According to the review of research evidence in Iran, this was the first study that specifically investigated caffeine consumption patterns and their relationship with the signs and symptoms of mental disorders. In addition, in this research, demographic variables have been widely studied, which can be very effective in the transparency of the issue.

However, the use of self-report tools and the cross-sectional nature of the present study are among the limitations of this research. Due to the increased costs, taking a blood test to measure caffeine accurately was impossible, so self-report scales alone may affect how people respond. Therefore, using other methods to obtain information from participants can help increase the generalizability and validity of the findings. This study was conducted during the outbreak of COVID-19, which may have affected the amount of caffeine consumption due to the remote working of many jobs. Also, since sampling was done in public places, many people may have been quarantined at home due to the spread of COVID-19. Since this research had examined a wide range of variables, if it was conducted online, due to its length, the possibility of completing the questionnaire was very low, so the research was conducted on pencil paper. However, when the questionnaire was administered, some people refused to answer because it was too long, and the number of returned questionnaires was incomplete. The exclusion of individuals aged 60 years and older may limit the generalizability of the findings to the broader population, and caution should be exercised in generalizing the results. This age-related exclusion was implemented to minimize potential biases associated with prevalent health conditions in the elderly.

## Conclusion

The high rate of CUD and CWSs in the present sample highlights the need for education and treatments related to caffeine consumption. However, it should be noted that although there are different types of caffeinated products, and there are also different expectations regarding the effects of these beverages, it seems that each of these products may have different roles in causing CUD^21^. Therefore, more research should be done on caffeinated products separately. In addition, demographic variables (such as gender and social economic status) and caffeine use patterns (time of use, number of servings, etc.) can determine the prevalence of CUD and CWSs and their relationship with another research.

## Data Availability

The data that support the findings of this study are available from the corresponding author upon reasonable request.

## References

[1] Crocq M-A (2022). Alcohol, nicotine, caffeine, and mental disorders. Dialogues Clin. Neurosci..

[2] Vital-Lopez FG, Ramakrishnan S, Doty TJ, Balkin TJ, Reifman J (2018). Caffeine dosing strategies to optimize alertness during sleep loss. J. Sleep Res..

[3] Smith AP (2013). Caffeine, extraversion and working memory. J. Psychopharmacol..

[4] Grgic J, Mikulic P, Schoenfeld BJ, Bishop DJ, Pedisic Z (2019). The influence of caffeine supplementation on resistance exercise: A review. Sports Med..

[5] Londzin P, Zamora M, Kąkol B, Taborek A, Folwarczna J (2021). Potential of caffeine in Alzheimer’s disease—a review of experimental studies. Nutrients.

[6] Ren X, Chen J-F (2020). Caffeine and Parkinson’s disease: multiple benefits and emerging mechanisms. Front. Neurosci..

[7] Jiang X, Zhang D, Jiang W (2014). Coffee and caffeine intake and incidence of type 2 diabetes mellitus: A meta-analysis of prospective studies. Eur. J. Nutr..

[8] Shi X, Xue W, Liang S, Zhao J, Zhang X (2016). Acute caffeine ingestion reduces insulin sensitivity in healthy subjects: A systematic review and meta-analysis. Nutr. J..

[9] Temple JL, Bernard C, Lipshultz SE, Czachor JD, Westphal JA, Mestre MA (2017). The safety of ingested caffeine: A comprehensive review. Front. Psychiatry.

[10] Pohler H (2010). Caffeine intoxication and addiction. J. Nurse Pract..

[11] Winston AP, Hardwick E, Jaberi N (2005). Neuropsychiatric effects of caffeine. Adv. Psychiatric Treat..

[12] Jee HJ, Lee SG, Bormate KJ, Jung Y-S (2020). Effect of caffeine consumption on the risk for neurological and psychiatric disorders: Sex differences in human. Nutrients.

[13] Sweeney MM, Weaver DC, Vincent KB, Arria AM, Griffiths RR (2020). Prevalence and correlates of caffeine use disorder symptoms among a United States sample. J. Caffeine Adenosine Res..

[14] Adeleye QA, Attama CM, Egbeobauwaye O, Angela O (2023). Psychosis following caffeine consumption in a young adolescent: Review of case and literature. Ann. Afr. Med..

[15] Lara DR (2010). Caffeine, mental health, and psychiatric disorders. J. Alzheimer's Dis..

[16] Tondo L, Rudas N (1991). The course of a seasonal bipolar disorder influenced by caffeine. J. Affect. Disord..

[17] Ogawa N, Ueki H (2003). Secondary mania caused by caffeine. Gen. Hosp. Psychiatry.

[18] Yamamoto T, Yoshizawa K, Kubo S, Emoto Y, Hara K, Waters B (2015). Autopsy report for a caffeine intoxication case and review of the current literature. J. Toxicol. Pathol..

[19] Mermi O, Kilic F, Gürok MG, Yilmaz S, Baykara S, Canan F (2016). Habitual caffeine use in psychiatric patients: relationship with sleep quality and symptom severity. Anatol. J. Psychiatry/Anadolu Psikiyatri Dergisi.

[20] Booth N, Saxton J, Rodda S (2020). Estimates of caffeine use disorder, caffeine withdrawal, harm and help-seeking in New Zealand: A cross-sectional survey. Addict. Behav..

[21] Ágoston C, Urbán R, Richman MJ, Demetrovics Z (2018). Caffeine use disorder: An item-response theory analysis of proposed DSM-5 criteria. Addict. Behav..

[22] Juliano LM, Huntley ED, Harrell PT, Westerman AT (2012). Development of the caffeine withdrawal symptom questionnaire: Caffeine withdrawal symptoms cluster into 7 factors. Drug Alcohol Dependence.

[23] Najarian B, Davoodi I. Construction and validation of a short form of the SCL-90-r (SCL-25). 2001.

[24] Derogatis LR, Rickels K, Rock AF (1976). The SCL-90 and the MMPI: A step in the validation of a new self-report scale. Br. J. Psychiatry.

[25] Smelser NJ, Baltes PB (2001). International Encyclopedia of the Social & Behavioral Sciences.

[26] Geifman N, Cohen R, Rubin E (2013). Redefining meaningful age groups in the context of disease. Age (Dordr)..

[27] Akhgar SM (2020). A brief look at the history, botany and preparation and production of various types of tea in Iran. Iran. Plant Biotechnol. Q..

[28] Benton D (2011). Lifetime Nutritional Influences on Cognition, Behaviour and Psychiatric illness.

[29] Frary CD, Johnson RK, Wang MQ (2005). Food sources and intakes of caffeine in the diets of persons in the United States. J. Am. Dietetic Assoc..

[30] Choi J (2020). Motivations influencing caffeine consumption behaviors among college students in Korea: Associations with sleep quality. Nutrients.

[31] Malinauskas BM, Aeby VG, Overton RF, Carpenter-Aeby T, Barber-Heidal K (2007). A survey of energy drink consumption patterns among college students. Nutr. J..

[32] Gallimberti L, Buja A, Chindamo S, Vinelli A, Lazzarin G, Terraneo A (2013). Energy drink consumption in children and early adolescents. Eur. J. Pediatrics.

[33] Mason MJ, Scammon DL (2011). Unintended consequences of health supplement information regulations: The importance of recognizing consumer motivations. J. Consum. Affairs.

[34] AlAteeq DA, Alotaibi R, Al Saqer R, Alharbi N, Alotaibi M, Musllet R (2021). Caffeine consumption, intoxication, and stress among female university students: A cross-sectional study. Middle East Curr. Psychiatry.

[35] Richards G, Smith A (2015). Caffeine consumption and self-assessed stress, anxiety, and depression in secondary school children. J. Psychopharmacol..

[36] Evans SM, Griffiths RR (1999). Caffeine withdrawal: A parametric analysis of caffeine dosing conditions. J. Pharmacol. Exp. Ther..

[37] Fredholm BB, Bättig K, Holmén J, Nehlig A, Zvartau EE (1999). Actions of caffeine in the brain with special reference to factors that contribute to its widespread use. Pharmacol. Rev..

[38] Lane JD (2011). Caffeine, glucose metabolism, and type 2 diabetes. J. Caffeine Res..

[39] Orbeta RL, Overpeck MD, Ramcharran D, Kogan MD, Ledsky R (2006). High caffeine intake in adolescents: associations with difficulty sleeping and feeling tired in the morning. J. Adolesc. Health.

[40] O’callaghan F, Muurlink O, Reid N (2018). Effects of caffeine on sleep quality and daytime functioning. Risk Manag. Healthcare Policy.

[41] Bergin JE, Kendler KS (2012). Common psychiatric disorders and caffeine use, tolerance, and withdrawal: An examination of shared genetic and environmental effects. Twin Res. Human Genetics.

[42] Hearn JK, Reiff T, McBride AB, Kelly MB (2020). Caffeine-induced psychosis and a review of statutory approaches to involuntary intoxication. J. Am. Acad. Psychiatry Law.

